# Retroperitoneal Hemorrhage After Percutaneous Posterior Spinal Instrumentation: A Case Report

**DOI:** 10.1155/cro/2550320

**Published:** 2026-10-14

**Authors:** Jeremy A. Bernhardt, Santaram Vallurupalli, Sean Duguay

**Affiliations:** ^1^ Department of Orthopedic Surgery and Rehabilitation University of Oklahoma College of Medicine Oklahoma City Oklahoma USA jsd.js.cn; ^2^ Department of Interventional Radiology University of Oklahoma College of Medicine Oklahoma City Oklahoma USA jsd.js.cn

**Keywords:** case report, embolization, iliolumbar artery, retroperitoneal hemorrhage

## Abstract

Retroperitoneal hemorrhages are an uncommon but potentially fatal complication in the setting of recent spine surgery. Patients generally report lower abdominal or groin pain, and they may demonstrate signs of hemodynamic instability. Prompt recognition of retroperitoneal hemorrhages requires a thorough history and physical examination, monitoring of appropriate laboratory values, and often advanced imaging. Whereas treatment can occasionally be achieved with conservative measures, retroperitoneal hemorrhage is most commonly treated with embolization. We present a case of spontaneous retroperitoneal hemorrhage after percutaneous L3 to pelvis posterior spinal instrumentation and L4–L5 posterolateral fusion with allograft. In our case, the patient was noted to be hypotensive and began reporting right lower quadrant abdominal pain on postoperative Day 2. Investigation with computed tomography angiography revealed a right retroperitoneal hematoma with small areas of active bleeding, ultimately requiring an embolization procedure with interventional radiology. The patient was last seen for a 1‐year follow‐up, at which time he demonstrated 5/5 motor strength and fully intact sensation throughout L2–S1 of the bilateral lower extremities. He complained of mild right lumbar pain over the region of hardware after recently losing approximately 30 pounds and recent balance issues. This case highlights common presenting signs/symptoms of a relatively uncommon and potentially fatal complication in the setting of percutaneous spinal surgery; we hope to increase awareness and improve prompt recognition among providers to help minimize serious complications for patients.

## 1. Introduction

Spine surgery has continued progressing toward minimally invasive techniques, including percutaneous posterior spinal instrumentation. However, percutaneous surgery is not without risk. Our case study discusses an uncommon complication, retroperitoneal hemorrhage (RH).

Spontaneous retroperitoneal hemorrhages (SRHs) have been reported in association with antiplatelet and anticoagulation therapy, arterial lesions, hemodialysis, and coagulopathies [1, 2]. There are case reports indicating enoxaparin, a commonly used anticoagulant in orthopedic surgery, as a cause of large and even fatal RH [3, 4]. Recently, several case reports have pointed to iatrogenic lumbar artery injury as an additional cause [5–9].

As SRH is an infrequent but potentially fatal complication, it is important to be aware of concerning signs and symptoms. The most common complaint is sudden onset of lower abdominal or groin pain. These patients can demonstrate signs of hypovolemic shock secondary to massive hemorrhage. Prompt recognition can allow the patient to be treated with embolization [10]. Here, we present the case of an individual with an SRH after percutaneous L3 to pelvis posterior spinal instrumentation and L4–L5 posterolateral fusion with allograft.

While reports do exist, there remains a relative paucity of information regarding the occurrence of SRH after spinal surgery. In reporting this case, our aim is to increase awareness among healthcare providers, aid in expeditious recognition, and help limit potentially serious complications.

## 2. Case Presentation

The patient involved in the following case gave oral consent for publication. This case report is exempt from IRB approval according to the policy of The University of Oklahoma Health Sciences Center. The authors adhered to the CARE guidelines (File S1) and have provided a timeline (Table 1). A gentleman in his 40s presented with 2 months of subjective weakness and urinary incontinence. Relevant past medical history included paroxysmal atrial fibrillation (for which he was taking rivaroxaban 20 mg once daily) and Type 2 diabetes mellitus. Known drug allergies included trimethoprim–sulfamethoxazole and penicillins. He reported regular tobacco use (0.25 cigarette packs/day for 3 years) and alcohol dependence (2–3 pints/day). He did not report any family history of medical issues. During initial presentation, he was treated with intravenous antibiotics for T6–T7 discitis. Three weeks after discharge, the patient returned with lumbar pain. Inflammatory markers were obtained on the day of repeat admission. His C‐reactive protein was 113.2 mg/L, erythrocyte sedimentation rate was 84 mm/h, and procalcitonin was 0.23 ng/mL. Physical exam revealed tenderness to palpation over the midline lumbar spine, endorsement of pain with bilateral straight leg raise, 5/5 motor strength and full sensation L2–S1 in bilateral lower extremities, and no upper motor neuron signs. The patient′s physical examination and inflammatory markers prompted workup with a magnetic resonance image (MRI) with contrast of the lumbar spine on Day 1 of admission. This revealed L4–L5 discitis, L4 and L5 osteomyelitis, and dynamic instability at L4/L5 with bilateral pars fractures. He subsequently underwent percutaneous L3 to pelvis posterior spinal instrumentation and L4–L5 posterolateral fusion with allograft on Day 4 of admission. Tissue samples were taken for culture during the procedure and sent for aerobic, anaerobic, fungal, and acid‐fast bacillus testing. None of these samples demonstrated any growth during their period of observation. Due to a history of paroxysmal atrial fibrillation, the patient had been taking rivaroxaban prior to admission as previously noted. He was transitioned to therapeutic enoxaparin (150 mg twice daily, which was approximately 1 mg/kg) immediately upon admission, and his morning dose was held on the day of surgery. He sustained an estimated 100 mL of intraoperative blood loss, and there were no intraoperative concerns regarding the patient′s hemodynamic stability. The enoxaparin was resumed with his evening dose on postop Day 1.

**Table 1:** Timeline of key events.

January 1, 2024	Original admission for thoracic osteomyelitis
February 6, 2024	Readmitted with new‐onset low back pain. Found to have L4/L5 osteomyelitis
February 12, 2024	Underwent irrigation and debridement, posterior spinal fusion, and instrumentation from L3–S1
February 13, 2024	Anticoagulation resumed evening of postop Day 1
February 14, 2024	Retroperitoneal hemorrhage discovered. Underwent embolization with interventional radiology. Transferred to MICU and started on norepinephrine
February 15, 2024	Norepinephrine discontinued
February 16, 2024	Transferred back to medical floor bed
February 23, 2024	Anticoagulation held until this point. Apixaban was started on this day. Discharged to inpatient rehab

Of note, the patient′s preoperative hemoglobin was 104 g/L, and his postop Day 1 hemoglobin was 96 g/L. On postop Day 2, he became hypotensive and reported right lower quadrant abdominal pain. A point‐of‐care hemoglobin was 60 g/L. At this time, all anticoagulation was held. His platelet count was within normal limits throughout the preoperative and postoperative period (range 230–300 × 10^3^/*μ*L). Postoperative coagulation studies demonstrated prothrombin time (PT) of 15.9 s, partial thromboplastin time (PTT) of 31.2 s, and international normalized ratio (INR) of 1.4.

At this point, our differential diagnosis included surgical site bleed, vascular injury, or other occult bleed. Upon examination, the patient′s surgical site was noted to be benign; however, he was reporting abdominal pain. Due to increased suspicion for intra‐abdominal pathology, he was taken for emergent computed tomography angiography (CTA) of the abdomen/pelvis. This revealed a 15 cm right retroperitoneal hematoma with small areas of active bleeding inferiorly along the right psoas (Figures 1 and 2). The CTA did not demonstrate any screws violating the anterior cortex of vertebral bodies, nor was there any concern for violation intraoperatively. He was taken emergently for coil embolization of the right L4 lumbar artery, right iliolumbar artery, and a branch from the right external iliac artery (Figure 3A,B). His care was transitioned to the medical intensive care unit (ICU) prior to embolization, and he remained there for 48 h, including a 24‐h period during which he required norepinephrine. He was then transferred back to the floor where the remainder of his stay was uneventful. The patient was monitored for any sign of recurrence with daily examinations, and a heparin/anti‐Xa level was regularly checked while inpatient. Anticoagulation was held until apixaban was given prior to discharge on postop Day 11. There was no attempted anticoagulation reversal at any point during his treatment course.

**Figure 1:**
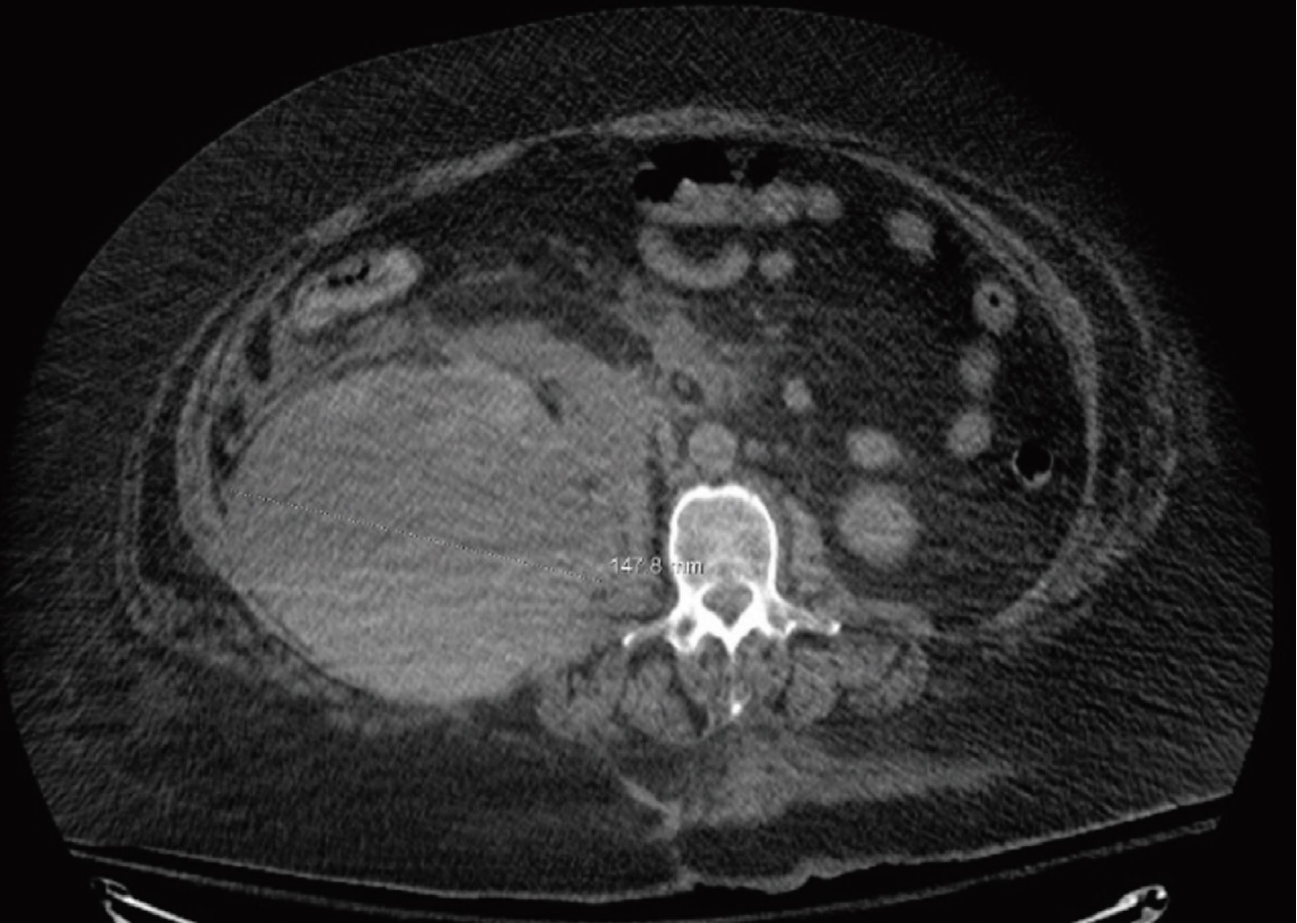
CT pelvis, axial image. Right retroperitoneal hematoma on CTA abdomen/pelvis.

**Figure 2:**
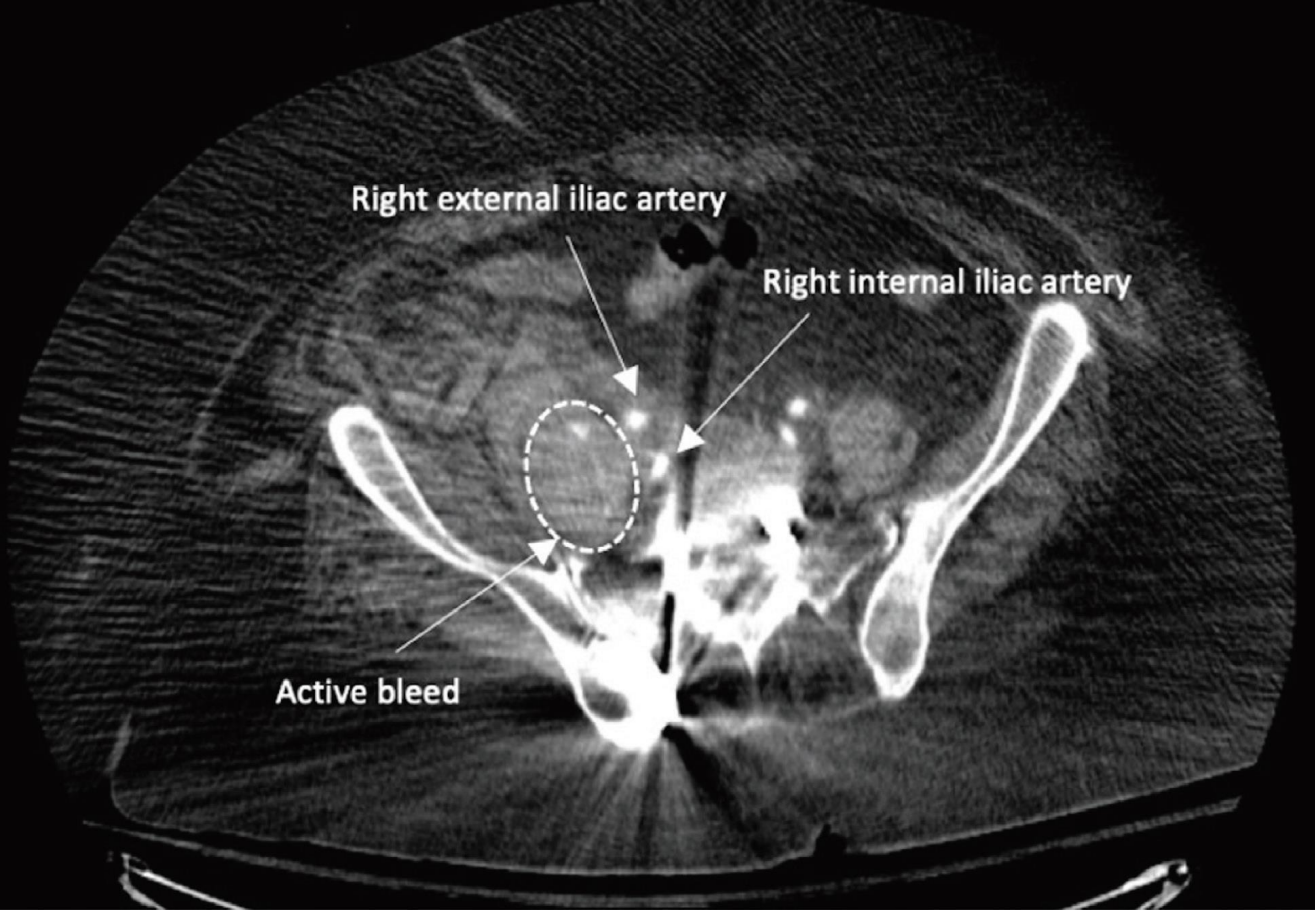
CT pelvis, axial image, arterial phase showing contrast extravasation, indicating an active arterial bleed.

**Figure 3:**
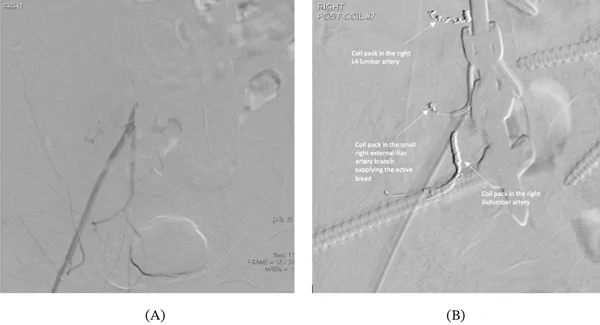
(A) Right external iliac arteriogram demonstrating active arterial bleeding from a small proximal branch. (B) Postcoil embolization fluoroscopic imaging of the multiple coil packs.

Patient was last seen at approximately 1 year postoperatively. At that time, he demonstrated fully intact sensorimotor function throughout his bilateral lower extremities. He reported concern secondary to right lumbar hardware prominence after recent weight loss, and he expressed issues with his balance which was starting to limit his function. Workup was ordered for evaluation of cervical myelopathy versus Wernicke–Korsakoff syndrome, but the patient has not returned for further discussion, limiting some potentially useful final outcome data.

## 3. Discussion

An RH, whether spontaneous or iatrogenic, can be a medical emergency. As this is a relatively unexpected complication in the postoperative period of a spine surgery, the provider must be alert to potential warning signs and symptoms. This has been referred to as Lenk′s triad which includes acute onset of abdominal or flank pain, a palpable mass on abdominal exam, and symptoms of hypovolemic shock and clinical deterioration [2]. Early detection is critical, as prompt intervention with embolization can stabilize the patient and avert further problems [10]. CT is considered the gold standard for diagnosis [11].

Several cases of spontaneous and iatrogenic retroperitoneal bleeds have been reported in the literature. Pathi et al. presented four cases associated with either anticoagulation or coagulopathy [12]. Three patients were taking warfarin chronically and had recently started receiving supplemental treatment with enoxaparin. The fourth patient was being anticoagulated for a coagulopathy in the setting of liver failure. All of the patients presented with abdominal/groin pain and hemodynamic instability. They had confirmed hemorrhage on CTA that was successfully controlled via embolization. The identification of enoxaparin as an underlying risk factor is particularly pertinent to orthopedic surgeons considering its widespread use in postoperative DVT prophylaxis. Enoxaparin has been indicated as a potential pharmacological cause of RH in multiple settings, including the treatment of myocardial infarctions [4] and routine orthopedic aftercare [3]. Renal patients have been identified as another higher risk patient population. Kim et al. reviewed 10 case reports including 14 total patients that experienced massive hemoperitoneum secondary to spontaneous lumbar artery rupture [10]. Among these patients, nine were undergoing dialysis therapy, whereas the remaining five were receiving either antiplatelet or anticoagulation therapy. These cases highlight the need for increased attentiveness in patients with coagulopathies or taking antiplatelet/anticoagulation agents.

In addition to underlying medical conditions and active medications, spine surgeons should also be mindful of SRHs as a potential postoperative complication. Both larger open approaches and less invasive open approaches have been implicated as causes of iatrogenic RH [7, 13]. Although iatrogenic retroperitoneal hematomas are more common with anterior or lateral interbody approaches, RHs should be considered in posterior‐alone patients due to complications from instrumentation or discectomies, as there have been cases cited in the literature. Ahn et al. and Cho et al. each reported postoperative retroperitoneal hematomas following transforaminal percutaneous endoscopic lumbar discectomy [5, 6]. It is likely that the segmental artery branch around the neural foramen is at risk of injury during this procedure due to its typical anatomic location. A high index of suspicion should be maintained in the postoperative setting with patients complaining of abdominal and/or groin pain, especially if there is any associated hemodynamic concern or substantial change in hemoglobin.

Whereas there is a lack of Level I evidence regarding the best treatment option for RHs, arterial embolization with interventional radiology is largely considered to be the treatment of choice [1]. This is especially true in cases of hemodynamic instability or identification of active bleed foci with arteriography. Conservative management has been proposed as a viable option in patients without hemodynamic compromise. This generally includes a combination of anticoagulation reversal, volume resuscitation, and transfusion of blood products (our facility generally utilizes a hemoglobin of 70.0 g/L as a threshold for transfusion) [3, 14]. Serial monitoring is critical in these cases to ensure a more direct intervention does not become necessary. Open surgery is usually only considered in patients when interventional radiology treatment is unsuccessful [1].

Our case adds to the literature by demonstrating an unexpected postoperative course following a relatively routine procedure. After initially being stable for more than 24 h postoperatively, our patient began reporting abdominal pain. At that point, he was noted to have a hemoglobin of 60 g/L, hemodynamic instability, and active bleeding foci identified by arteriography. He received two units of PRBCs, and he was treated successfully with coil embolization. The exact cause of the retroperitoneal bleed was never certain. We hypothesize that the patient taking rivaroxaban could have led to an anticoagulant‐induced SRH secondary to vessel fragility. However, due to the proximity of the bleed to our surgical intervention, it is also possible that the hemorrhage was iatrogenic in nature.

This case report demonstrates that a retroperitoneal bleed can occur in the early postoperative period after percutaneous spinal surgery. Special care should be taken when a patient is known to be at higher risk secondary to recent anticoagulation use, a history of a coagulopathy, or is planned for instrumentation near segmental vessels. Early recognition of common signs/symptoms is critical for prompt treatment and prevention of a potentially fatal hemorrhage.

## 4. Conclusion

RH after percutaneous spinal surgery is a rare, but potentially fatal, complication. A high index of suspicion must be maintained by the provider when a postoperative patient is reporting abdominal pain.

## Author Contributions

Jeremy A. Bernhardt: reviewed pertinent history and prepared/edited manuscript; Santaram Vallurupalli: manuscript editing; Sean Duguay: provided embolization images.

## Funding

No funding was received for this manuscript.

## Conflicts of Interest

The authors declare no conflicts of interest.

## Supporting information


**Supporting Information** Additional supporting information can be found online in the Supporting Information section. File S1: CARE checklist demonstrating adherence to the CARE guidelines.

## Data Availability

Data sharing is not applicable to this article as no datasets were generated or analyzed during the current study.
