# Supplementary material for: Retroperitoneal Hemorrhage After Percutaneous Posterior Spinal Instrumentation: A Case Report
Source: Case Rep Orthop. 2026 Oct 14;2026:2550320. doi: 10.1155/cro/2550320 (PMC13662823; doi:10.1155/cro/2550320)
Supplement: Supplementary file 1 [file CRO-2026-2550320-s001.pdf]

CARE case report guidelines

CARE Checklist of information to include when writing a case report

(cc) BY-NC-ND

Topic	Item	Checklist item description	Reported on Line
**Title**	1	The diagnosis or intervention of primary focus followed by the words "case report"	3-4
**Key Words**	2	2 to 5 key words that identify diagnoses or interventions in this case report, including "case report"	64
**Abstract (no references)**	3a	Introduction: What is unique about this case and what does it add to the scientific literature?	58-60
	3b	Main symptoms and/or important clinical findings	52-53
	3c	The main diagnoses, therapeutic interventions, and outcomes	53-55
	3d	Conclusion—What is the main "take-away" lesson(s) from this case?	58-62
**Introduction**	4	One or two paragraphs summarizing why this case is unique (**may include references**)	69-70, 83-86
**Patient Information**	5a	De-identified patient specific information.	90
	5b	Primary concerns and symptoms of the patient.	90-91, 97
	5c	Medical, family, and psycho-social history including relevant genetic information	91-95
	5d	Relevant past interventions with outcomes	95-96
**Clinical Findings**	6	Describe significant physical examination (PE) and important clinical findings.	116-117
**Timeline**	7	Historical and current information from this episode of care organized as a timeline	Yes
**Diagnostic Assessment**	8a	Diagnostic testing (such as PE, laboratory testing, imaging, surveys).	117-121, 124-125
	8b	Diagnostic challenges (such as access to testing, financial, or cultural)	N/A
	8c	Diagnosis (including other diagnoses considered)	197-200
	8d	Prognosis (such as staging in oncology) where applicable	N/A
**Therapeutic Intervention**	9a	Types of therapeutic intervention (such as pharmacologic, surgical, preventive, self-care)	128-135
	9b	Administration of therapeutic intervention (such as dosage, strength, duration)	135
	9c	Changes in therapeutic intervention (with rationale)	135-137
**Follow-up and Outcomes**	10a	Clinician and patient-assessed outcomes (if available)	138-141
	10b	Important follow-up diagnostic and other test results	141-143
	10c	Intervention adherence and tolerability (How was this assessed?)	N/A
	10d	Adverse and unanticipated events	N/A
**Discussion**	11a	A scientific discussion of the strengths AND limitations associated with this case report	143
	11b	Discussion of the relevant medical literature **with references**.	145-191
	11c	The scientific rationale for any conclusions (including assessment of possible causes)	197-200
	11d	The primary "take-away" lessons of this case report (without references) in a one paragraph conclusion	208-210
**Patient Perspective**	12	The patient should share their perspective in one to two paragraphs on the treatment(s) they received	
**Informed Consent**	13	Did the patient give informed consent? Please provide if requested	Yes [x] No [ ]
